# Telepsychiatry: the use of technology to improve access to mental health care

**DOI:** 10.1192/j.eurpsy.2023.1114

**Published:** 2023-07-19

**Authors:** M. Palazzo, D. Conti, A. Galbassini, T. G. Prodi, M. Cerioli, D. Bernardo

**Affiliations:** 1 Department of Mental Health and Addiction, ASST FBF Sacco; 2 Department of Mental Health, Department of Biomedical and Clinical Sciences Luigi Sacco, University of Milan, ASST FBF-Sacco, Milano, Italy

## Abstract

**Introduction:**

Telepsychiatry (TP) uses communication technology to provide psychiatric consultation to patients unable to reach consultation services. Due to COVID-19 outbreak, many mental health services implemented TP. The University of Milan developed a patient-specialist video consultation service: the Cure Ospedaliere Domiciliari (Home Hospital Care system; COD20).

**Objectives:**

The aim of the study was to assess the digital skills of the mental health professionals and to assess both the confidence and the satisfaction with the COD20 platform, as well as their skills in handling certain degrees of technostress.

**Methods:**

Mental health professionals of the outpatient clinics of the department were interviewed through an online anonymous survey. Data collected were sociodemographic, job position, educational level, digital skills, adequacy of devices in the workplace, satisfaction degree, ease of use of the COD20 tool, as well as main technostress score. Data were analyzed using SPSS v.27.

**Results:**

Among 95 subjects, more than 95% of the sample is familiar with the use of electronic devices, such as smartphones, tablets, and computers; 93% employs appropriate devices in the workplace. Only 12% had an ECDL certificate, while the majority of the sample (77%) learnt the use of electronic devices independently. The levels of the digital skills were considered intermediate-advanced for communication and information research. Despite all the respondents being aware of the use of COD20, only 50% received adequate training; 77% deemed it worthwhile to attend an individual or a group training (40% vs 43%). Telemedicine was used for clinical interviews by 80% of the sample: 41% of these used Telemedicine at least 10 times/year, 18% between 10 and 20 times/year, and 42% more than 20 times/year. With regard to the appreciation of the COD20 platform, 75% of the sample considered this tool useful, while 61% considered it easy to use. There is a significant correlation between the ease of use and a higher level of education (p<0,00). Among all categories, psychologists were more likely to use the platform compared to other workers ( p=0,016). The average score of technostress among operators was 22.78±6.84 (maximum score: 45).

**Image:**

**Figure:**
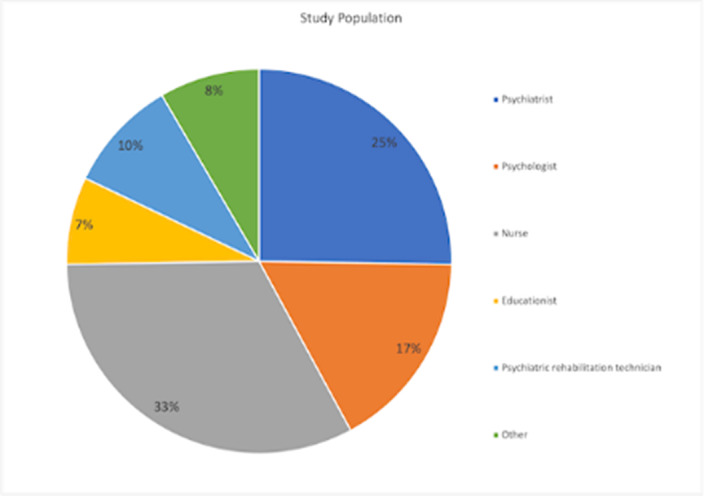

**Image 2:**

**Figure:**
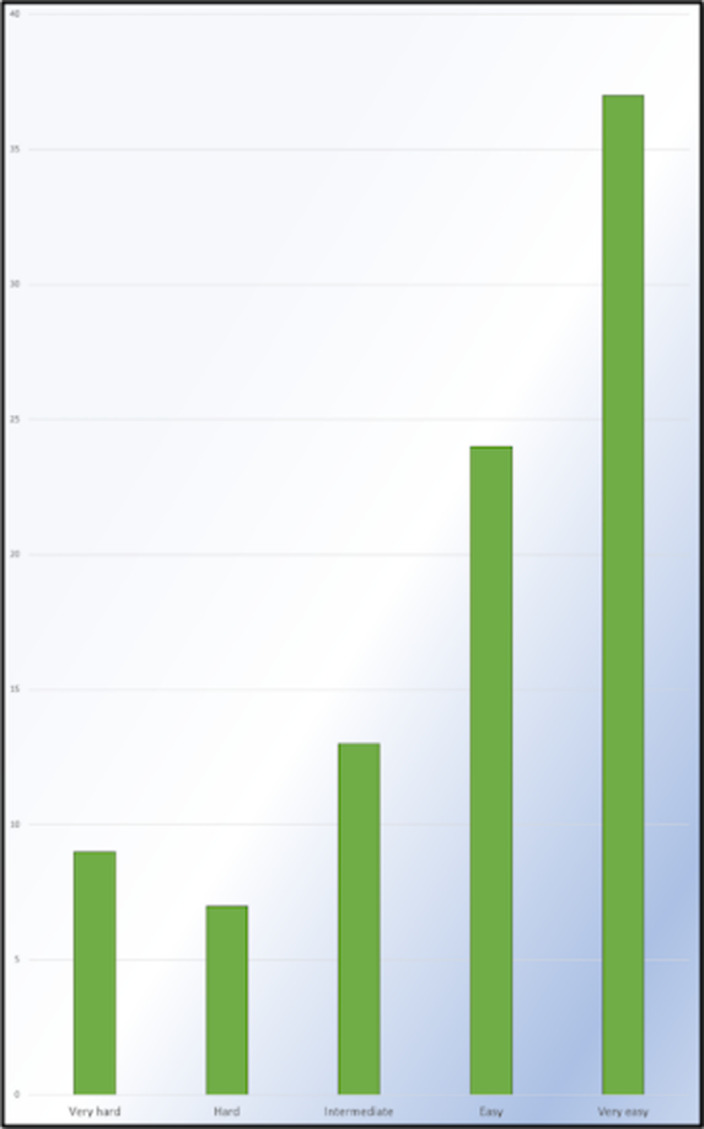

**Conclusions:**

TP can improve mental health professionals’ working conditions. The COD20 platform represents a valid implementation in mental health care. It is necessary to provide training and updated programs for healthcare workers in order to facilitate the use of TP tools.

**Disclosure of Interest:**

None Declared

